# CDCP1 Identifies a CD146 Negative Subset of Marrow Fibroblasts Involved with Cytokine Production

**DOI:** 10.1371/journal.pone.0109304

**Published:** 2014-10-02

**Authors:** Mineo Iwata, Beverly Torok-Storb, Elizabeth A. Wayner, William G. Carter

**Affiliations:** Fred Hutchinson Cancer Research Center, Seattle, Washington, United States of America; National Institutes of Health, United States of America

## Abstract

In vitro expanded bone marrow stromal cells contain at least two populations of fibroblasts, a CD146/MCAM positive population, previously reported to be critical for establishing the stem cell niche and a CD146-negative population that expresses CUB domain-containing protein 1 (CDCP1)/CD318. Immunohistochemistry of marrow biopsies shows that clusters of CDCP1+ cells are present in discrete areas distinct from areas of fibroblasts expressing CD146. Using a stromal cell line, HS5, which approximates primary CDCP1+ stromal cells, we show that binding of an activating antibody against CDCP1 results in tyrosine-phosphorylation of CDCP1, paralleled by phosphorylation of Src Family Kinases (SFKs) Protein Kinase C delta (PKC-δ). When CDCP1 expression is knocked-down by siRNA, the expression and secretion of myelopoietic cytokines is increased. These data suggest CDCP1 expression can be used to identify a subset of marrow fibroblasts functionally distinct from CD146+ fibroblasts. Furthermore the CDCP1 protein may contribute to the defining function of these cells by regulating cytokine expression.

## Introduction

Human marrow stromal cells are non-hematopoietic mesenchymal cells that can be cultured from aspirated marrow and expanded in vitro. In vivo they constitute the relatively static elements of the marrow microenvironment (ME). In vitro they express membrane molecules and secreted factors reported to play a role in regulating the maintenance, expansion, and differentiation of hematopoietic stem and progenitor cells. Contained within the in vitro expanded population are precursors for a variety of tissues including fibroblasts, endothelial cells, bone and cartilage [1]. Expanded marrow stromal cells have been extensively studied as potential tools in regenerative medicine, however the in vivo effects of infused stromal cells are not consistent [2]–[4]. It is hypothesized that this is due to qualitative differences among cell preparations [5]–[9].

Several immunophenotypes from various human and mouse stromal cell preparations have been studied in an attempt to identify functionally relevant cell subsets and their progenitors. CD146/MCAM [10], CD271/Low affinity NGFR [11], mKirrel3 [12] and CD105+/SSEA3+ (Muse cells) [13] were proposed as cell surface marker molecules for the relevant human population. CD105+/CD90- cells [14], Nestin+ cells [15], CXCL12/SDF1+ cells (CAR cells) [16], Mx1+ cells [17], NG/CSPG4+ cells [18], LepR+ cells [19], and ENPEP+ cells [20] were reported as mouse stromal cells that help maintain hematopoiesis. Currently the association between the various subsets defined by immunophenotype and specific ME function is not clear [3]. Furthermore, a defining function for the marker molecules, such as a ligand to the CD146 adhesion molecule or even a ligand to the hematopoietic stem/progenitor marker CD34, has not been identified.

Our effort to functionally define ME niches has focused on immortalizing and cloning functionally distinct non-hematopoietic cells present in primary human marrow long-term cultures [21]. Previously we have reported extensively on two lines designated HS5 and HS27a which differ in phenotype and function: HS5 is negative for CD146/MCAM and secretes growth factors leading to the proliferation and differentiation of CD34+ hematopoietic stem/progenitor cells, whereas HS27a is positive for CD146 and expresses activities associated with the stem cell niche [21], [22]. Despite these differences both cell lines were shown by DNase I hypersensitive site mapping to be closely related to marrow fibroblasts but not endothelial cells [22]. While CD146 positive cells have been identified in human marrow, the identification of HS5-like stromal cells in vivo has been difficult due to lack of marker molecules uniquely expressed by the CD146-negative stromal cells.

In the present study, we report that CUB domain-containing protein 1 (CDCP1)/CD318 is uniquely expressed on the cell surface of CD146-negative primary marrow stromal cells and in HS5 cells. *In vivo* relevance is suggested by immunohistochemical detection in bone marrow biopsies of discrete areas of CDCP1+ stromal cells. CDCP1 is active and transduces signals through Src Family Kinases (SFKs) and Protein Kinase C *delta* (PKC-δ) upon stimulation by an activating antibody. Finally, knock-down experiments suggest that CDCP1 plays a role in regulating hematopoiesis-related cytokine expression.

## Materials and Methods

### Marrow and peripheral blood cells from normal donors

A protocol and consent form for obtaining de-identified samples of normal blood and bone marrow for the purpose of studying cellular functions that regulate hematopoiesis as described in this study has been approved by the Fred Hutchinson Cancer Research Center (FHCRC) Institutional Review Board (IR File# 314; Protocol 211:00). Informed consent is obtained by attending physicians in the outpatient service of the Seattle Cancer Care Alliance (SCCA).

Bone marrow aspirates were obtained from five healthy donors, and long-term marrow cultures were established: Donor #1, 25-year-old female; Donor #2, 51-year-old male; Donor #3, 54-year-old female; Donor #4, 40-year-old male; and Donor #5, 46-year-old male.

Bone marrow biopsies for immunohistochemistry were obtained from the posterior iliac crest from healthy donors at the SCCA and from surgical sites during joint replacement surgeries at the University of Washington Medical Center.

### Culture of marrow stromal cells

Long-term marrow cultures were established as modified Dexter cultures using buffy-coat cells grown in medium containing Iscove's Modified Dulbecco's Medium, 12.5% horse serum, 12.5% fetal calf serum (FCS), hydrocortisone sodium succinate, and sodium pyruvate as previously reported [23]. Thirty immortalized stromal cell clones were isolated from the Dexter-like culture as described [21]. Two of these, designated HS5 and HS27a, were used in this study. These cell lines are available through American Tissue Culture Collection (ATCC). HS5 cells were maintained in complete media [supplemented RPMI 1640 medium containing 10% FCS (HyClone, Logan, UT), L-glutamine and sodium pyruvate].

Marrow mononuclear cells were used to establish primary cultures of marrow stromal cells using a Lineage Cell Depletion Kit and magnetic separation (Miltenyi Biotec, Auburn, CA) according to the manufacturer's protocol. The cells were cultured in MSC Basal media with MSC stimulatory supplement according to the manufacturer's protocol (ALLCELLS, Emeryville, CA). The cells were passaged until the desired number of cells was reached (less than 8 passages). Absence of adherent hematopoietic cells such as macrophages was confirmed by flow cytometry using antibodies against CD45, CD14 and CD11c.

In some experiments, HS5 cells were stimulated with P3D9 monoclonal antibodies against CDCP1 [24], [25] for 10–30 minutes. IL-1b (2 ng/mL) and IFN gamma (1 ng/mL) were also used to stimulate HS27a and HS5 cells.

### Syber green real-time reverse transcription polymerase chain reaction (RT-PCR) analysis

Total RNA was purified using RNeasy spin columns (Qiagen, Valencia, CA), and then DNase-treated at 37°C for 30 min using 1000 U of RQ1 RNase-free DNase (Promega, Madison, WI). Samples were then reverse transcribed into cDNA with an oligo dT_12–18_ primer. Expression levels of RNA transcripts were quantitated by real-time RT-PCR performed using ABI Prism 7900HT Sequence Detector (Perkin Elmers, Boston, MA) as described before [26]. Forward and reverse oligonucleotide primers are as follows, in 5′ to 3′ orientation: CTAAATGGCGTGTACTGCAAGACC and TCTCAGGAGCCAGCAACTTGTCC for CDCP1; GGAAGGACTCATGACCACAGTCC and TCGCTGTTGAAGTCAGAGGAGACC for GAPDH. The data were represented by the average +/− SD for n≥2.

### Western blot analysis

HS5 and HS27a cells were cultured in 10 cm culture dish, and stimulated with and without P3D9 antibody for 30 min. After the cells were washed with cold PBS, proteins were extracted on ice in 1% Triton X-100 in phosphate-buffered saline (PBS) containing 0.5 mM oxidized sodium orthovanadate, 1 mM phenylmethylsulfonyl fluoride (PMSF), 50 mM sodium fluoride, and 2 mM N-ethylmaleimide (NEM) for 30 minutes. Extracted proteins were quantified by Fluorescamine (Sigma). After being heat-denatured in Laemmli sample buffer containing 5% β-mercaptoethanol, 60 µg of proteins were applied to sodium dodecyl sulfate-polyacrylamide gel electrophoresis (SDS-PAGE) with 10% acrylamide and immunoblotted to a nitrocellulose membrane. The membrane was blocked in Tris Balanced Salt Solution (TBS) containing 0.5% heat-denatured BSA and 0.1% Tween 20, and probed sequentially with 4G10 anti-phosphotyrosine monoclonal antibody and IRDye-conjugated secondary antibodies (Rockland). The bound fluorescence was visualized using Odyssey scanner (LI-COR) and quantitated using Odyssey Application Software. The membrane was re-probed with rabbit anti-CDCP1 or rabbit anti-phosphorylated PKC-δ (Y311) antibodies (Cell Signaling) and IRDye-conjugated anti-rabbit IgG secondary antibodies.

### Flow-based analysis of labeled cells

Primary stromal cells and HS5 cells were detached from culture plates by incubation at 37°C for 10 to 30 minutes in Cell Dissociation Buffer (non-enzymatic, Gibco, Grand Island, NY), and washed with Hank's Balanced Salt Solution (HBSS, Gibco). Non-specific binding to FcR was blocked by incubating in 10% FcR-blocking solution (Miltenyi Biotec) for >20 minutes at 4°C. Cells were then stained with PE-, APC-, or FITC-conjugated antibodies. Control staining was performed simultaneously using isotype-matched, irrelevant antibodies also directly conjugated to PE, APC or FITC. Cells were washed twice, and propidium iodide was added as a marker to exclude dead cells. The fluorescence intensity of the cells was analyzed by flow cytometry (FACSCalibur or FACSCanto; Becton Dickinson, San Jose, CA). The antibodies used are unconjugated anti-CDCP1 antibodies, FITC-conjugated anti-CDCP1 (BioLegend, San Diego CA), PE-conjugated anti-CD146 (BD Biosciences, Franklin ME) and FITC-conjugated antibodies against CD45 and CD14 (BD Biosciences). For the unconjugated antibodies, FITC-conjugated goat F(ab')_2_ anti-mouse IgG antibodies (Life Technologies, Grand Island, NY) were used as secondary antibodies.

### Immunofluorescence of CDCP1+ cells

HS5 cells were cultured in 8-chamber coverglass slides (Nalge Nunc, Naperville, IL) coated with 25 ng/mL of human fibronectin at 40,000 cells per chamber for 1 day. They were washed with PBS twice, fixed in 5% formalin in 0.1 M Sodium Cocadylate, pH 7.2, and 0.1 M Sucrose for 10 minutes at room temperature, and washed with PBS three times. The slides were permeabilized in PBS containing 0.5% Brij 98, 0.5 mM oxidized sodium orthovanadate, 1 mM phenylmethylsulfonyl fluoride (PMSF) and 1 mM N-ethylmaleimide (NEM) for 10 minutes. After rinsing with PBS twice, the slides were incubated with 1% heat-denatured BSA and 0.01% sodium azide for over 30 minutes to prevent nonspecific antibody binding. They were then incubated overnight with primary antibodies (Table S1 in File S1) or concentration and isotype-matched control immunoglobulins in the blocking solution. After washing with PBS, the slides were incubated with fluorochrome-conjugated secondary antibodies in the blocking media for 60 minutes in the dark. They were washed with PBS four times and stained with 4′6-diamidino-2-phenylindole (DAPI) at 1 µg/mL in PBS for 5 minutes. After a further wash with PBS, slides were mounted in PBS with 0.3% n-propyl gallate (Fluka) added as an anti-fade reagent, and the fluorescent staining was observed using a Deltavision Elite microscope (Applied Precision, Issaquah WA). An Olympus 60X/1.42 Plan Apo objective was used. Three dimensional image stacks were acquired on a Photometrics Coolsnap HQ cooled CCD camera, and deconvolved using a proprietary constrained iterative algorithm (Applied Precision). Volume rendered views of the image stacks were generated with Volocity software (Perkin Elmer, Waltham MA). Colocalization analysis between the green and red channels was performed on a representative area using softWoRx software (Applied Precision, Issaquah WA).

### Immunohistochemistry (IHC) of CDCP1+ cells in bone marrow

The Experimental Histopathology Laboratory at the FHCRC performed dual IHC to detect CDCP1+ cells in marrow biopsies. Bone marrow biopsies were immediately fixed in 10% neutral buffered formalin, decalcified in Formical 4 (Fisher Scientific, Pittsburgh PA), then processed and embedded in paraffin. Sections (5 µm) were deparaffinized and rehydrated to distilled water. Heat induced epitope retrieval was then performed with Trilogy (Cell Marque, CA) for 20 minutes in a steamer, followed by a 20-minute cool down. Slides were washed in Tris-based-saline containing 0.05% Tween20 (TBS-T), and incubated with 3% hydrogen peroxide for 8 minutes to block endogenous peroxidase activity. The sections were then incubated in TCT buffer (50 mM Tris-HCl, pH 7.6, 0.15 M NaCl, 0.25% (w/w) Casein and 0.1% Tween20) containing 15% human serum for 10 minutes to block non-specific binding for 10 minutes. They were incubated with the primary antibodies (anti-CDCP1; Cell Signaling Technology, Danvers, MA) for 1 hour. For a negative control, a concentration and isotype-matched control was used. Slides were washed, incubated with species specific polymer (PowerVision Rabbit HRP, Leica Biosystem, Buffalo Grove IL) for 30 minutes, and washed again. The slides were incubated further in DAB for 4 minutes twice, and counter-stained in 50% Tachas Hematoxylin. They were rinsed well with water and coversliped with Crystal mount. They were imaged using a Zeiss AXIO Imager Z2 microscope fitted with a 40X/0.75 Plan-Neofluar or a 100X/1.4 oil Plan-Apochromat objective lens (Carl Zeiss, Thornwood NY) and a Nikon E800 microscope with a 40X/0.95 PlanApo or a 100X/1.3 PlanApo oil immersion objective lens (Nikon). A Zeiss Axiocam MRc color CCD camera and Zeiss Axiovision acquisition software (Carl Zeiss) were used to acquire bright field images.

The specificity of the primary anti-CDCP1 antibody used in this study was confirmed by immunoblotting experiments using Knock-Down and Knock-In cells of CDCP1 (unpublished data by WGC). Many other primary antibodies against CDCP1 including in house and commercial monoclonal antibodies (clone CUB1, clone 309137 from R&D systems) and polyclonal antibodies (HPA010978 and HPA010979 from Sigma-Aldrich) were tried for paraffin embedded and decalcified bone marrow specimens. They all failed to bind to the antigens on the specimens or bound non-specifically.

### Transfection of HS5 cells with siRNA

HS5 cells were transfected with siRNA for CDCP1 (FlexiTube siRNA Hs_CDCP1__4, SI00341446, Qiagen, Hilden Germany; Stealth RNAiTM 10620312, Invitrogen), SDC1 (Hs_SDC1__1, SI00020587, Qiagen), KDM3B (Hs_JMJD1B__7, SI04270357, Qiagen), PKC-δ (Stealth RNAiTM 10620318, Invitrogen) or control siRNA (Luciferase GL2, Qiagen) using Lipofectamine RNAiMAX (Life Technologies, Grand Island NY) according to the manufacturer's protocol.

To avoid any off-target effects, four siRNAs for CDCP1 were tested, and we found that Hs_CDCP1_4 siRNA had no or minimum off-target effects compared to the other three siRNAs (Hs_CDCP1_2, SI00341432; HS_CDCP1_5, SI04170992;Hs_CDCP1_6, SI04199083, Qiagen).

### Phase-contrast microscopy

Images of the transfected cells were captured using a Nikon Eclipse Ti microscope with a 20X/0.45 lenz (Nikon, S Plan Fluor ELWD) and a DS-Qi1 camera (Nikon).

### Microarray hybridization and data analysis

Microarray hybridization and data analysis were conducted at the FHCRC Genomics Shared Resource. In brief, HS5 cells were transfected with siRNA for CDCP1, KDM3B, SDC1 or luciferase, and cultured for 2 days. Non-transfected HS5 cells were also used as a control. Total RNA was then extracted from the cells using RNeasy spin columns (Qiagen, Valencia, CA). Double-stranded cDNA and cRNA were synthesized from 200 ng of total RNA and hybridized to Illumina HumanHT-12 v4 Expression BeadChips (Illumina, San Diego, CA) using the manufacturer's standard protocols.

Microarray data was assessed for quality and quantile normalized using the Bioconductor package lumi [27]. Initial filtering included flagging probes that were below a signal “noise floor”, which was calculated as the 75th percentile of the negative control probe signals within each array. We subsequently filtered the dataset by employing a variance filter using the *shorth* function of the Bioconductor package genefilter. Differential gene expression was determined using luciferases as the common reference by employing the Bioconductor package limma [28], and a false discovery rate (FDR) method was used to correct for multiple testing [29].

Using a |log_2_(ratio)|≥1 with the false discovery rate (FDR) set to 5%, we identified a combined total of 573 probes as differentially expressed taken across all comparisons. The identified probes were the union of sig from the comparisons of CDCP1, KDM3B, SDC1 and non-transfection vs. luciferase as the common reference. Using this set of differentially expressed probes, we mean-centered each probe's normalized signal across all conditions and performed k-means clustering (Euclidean distance similarity matrix). Clustering and heat map presentation were performed using the TM4 suite module MultiExperimental Viewer (MeV) open source software [30]. Subcellular localization of the probe target genes was identified using Ingenuity Systems IPA software. (Ingenuity Systems, Redwood, CA).

### Cytokine analysis

Cytokine levels in the conditioned media were determined using enzyme-linked immunosorbent assays (ELISAs) using a Luminex200 multiplex assay instrument (Millipore, Billerica, MA), performed by the Cytokine Laboratory Shared Resource of the FHCRC. Antibodies against IL-8 were from Fisher Scientific (Pittsburgh, PA) and R&D Systems (Minneapolis, MN).

### Statistics

Means and SDs were measured, and statistically significant differences were identified by paired Student t test (*P*<0.05). Pearson correlation coefficients were calculated.

## Results

### CDCP1 is expressed in a minor subset of primary stromal cells and HS5 stromal cell line

**Figure 1:**
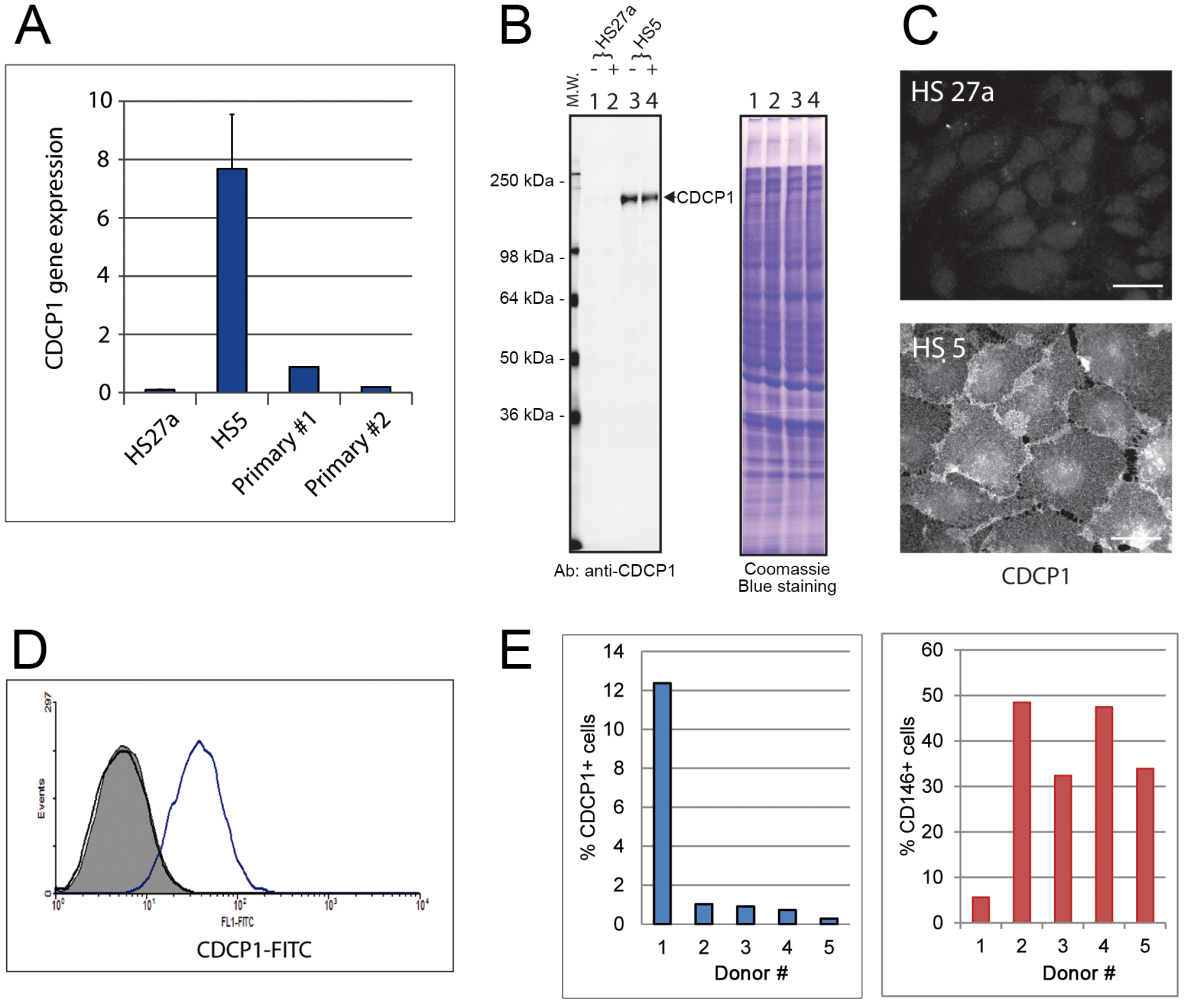
Expression of CDCP1 in bone marrow stromal cells. **Panel A**: Gene expression of CDCP1 in HS27a, HS5 and 2 primary stromal cultures was determined by qPCR. Y-axis shows relative gene expression of CDCP1. **Panel B**: Western blot (left panel) of Triton-X-100 extracts of HS27a and HS5 cells before (−) and after (+) P3D9 stimulation. Sixty micrograms of cellular proteins were loaded in each lane. Antibodies against CDCP1 were used for Western blotting. M.W. stands for molecular weight markers. Right panel shows protein staining with Coomassie Brilliant Blue to show the equal protein load. **Panel C**: Immunofluorescence staining of CDCP1 in HS27a cells (upper panel) and HS5 cells (lower panel). Scale bars, 20 µm. **Panel D**: Protein expression of CDCP1 on the surface of HS27a (black) and HS5 (blue) cells was determined by flow cytometry using P1C3 antibody against CDCP1. Flow cytometry using additional monoclonal anti-CDCP1 antibodies (CUB1, P5H10 and P3D9) which recognize different epitopes on CDCP1 showed the same results (shown in Figure S1 in File S1). Gray area within black line is the isotype-matched control. **Panel E**: Proportion of CDCP1+ and CD146+ stromal cells in primary marrow cultures. Bone marrow mononuclear cells were isolated from five healthy donors and primary long-term stromal cultures were established. The cells were stained with antibodies against CD146, CDCP1, CD14 and CD45, and were analyzed by flow cytometry. The proportion of CDCP1+ (left panel) and CD146+ (right panel) stromal cells are shown on the y-axis.

Global gene expression profiling was used to identify cell surface markers that distinguish between CD146-negative HS5 cells and CD146-positive HS27a stromal cells [31]. One cell surface molecule, CDCP1, was expressed exclusively in HS5. The microarray data of CDCP1 expression in the stromal cell lines was confirmed by quantitative PCR (Figure 1A), which was also used to confirm expression in primary stromal cells. Low but significant expression of CDCP1 was detected in primary stromal cells from two healthy donors, although the levels were variable between the donors. Western blot analysis and immunofluorescence staining confirm that CDCP1 protein is translated in HS5 cells (Figure 1B and C), whereas HS27a cells did not express CDCP1 protein, confirming the gene expression data. Flow cytometry of CDCP1 in the stromal cell lines shows that CDCP1 proteins were expressed on the surface of HS5 cells after translation (Figure 1D and Figure S1 in File S1). The proportion of CDCP1+ cells in primary marrow stromal cultures was analyzed by flow cytometry, and found to be variable among healthy donors (Figure 1E). We have estimated approximately 50% of the primary cells are negative for both CD146 and CDCP1. Many of the double-negative cells do express ALCAM/CD166. However, CD166 expression is not restricted to the double-negative population (data not shown). These data suggest that the primary cells display extensive heterogeneity for multiple markers that include a CDCP1+ subpopulation.

Although HS27a cells are CDCP1-negative at the resting state, we asked if the expression of CDCP1 could be induced after stimulation of the cells. IL-1β and interferon gamma (IFN⌈) are known potent stimulators of HS27a cells. The expression of several genes such as SDF-1, ICAM1, HLA-DR and PD-L1 (B7 homolog 1) increases in HS27a cells after incubation with IL-1β or IFN⌈ [32]–[34]. However, the expression of CDCP1 was not induced in HS27a cells after IL-1β or IFN⌈ stimulation (Figure S2 in File S1). These data suggest that CDCP1 is a reliable marker for HS5 cells, and a subpopulation of primary stromal cells.

### Stromal CDCP1 is active and transduces signals through Src-family kinase and PKC-δ

**Figure 2:**
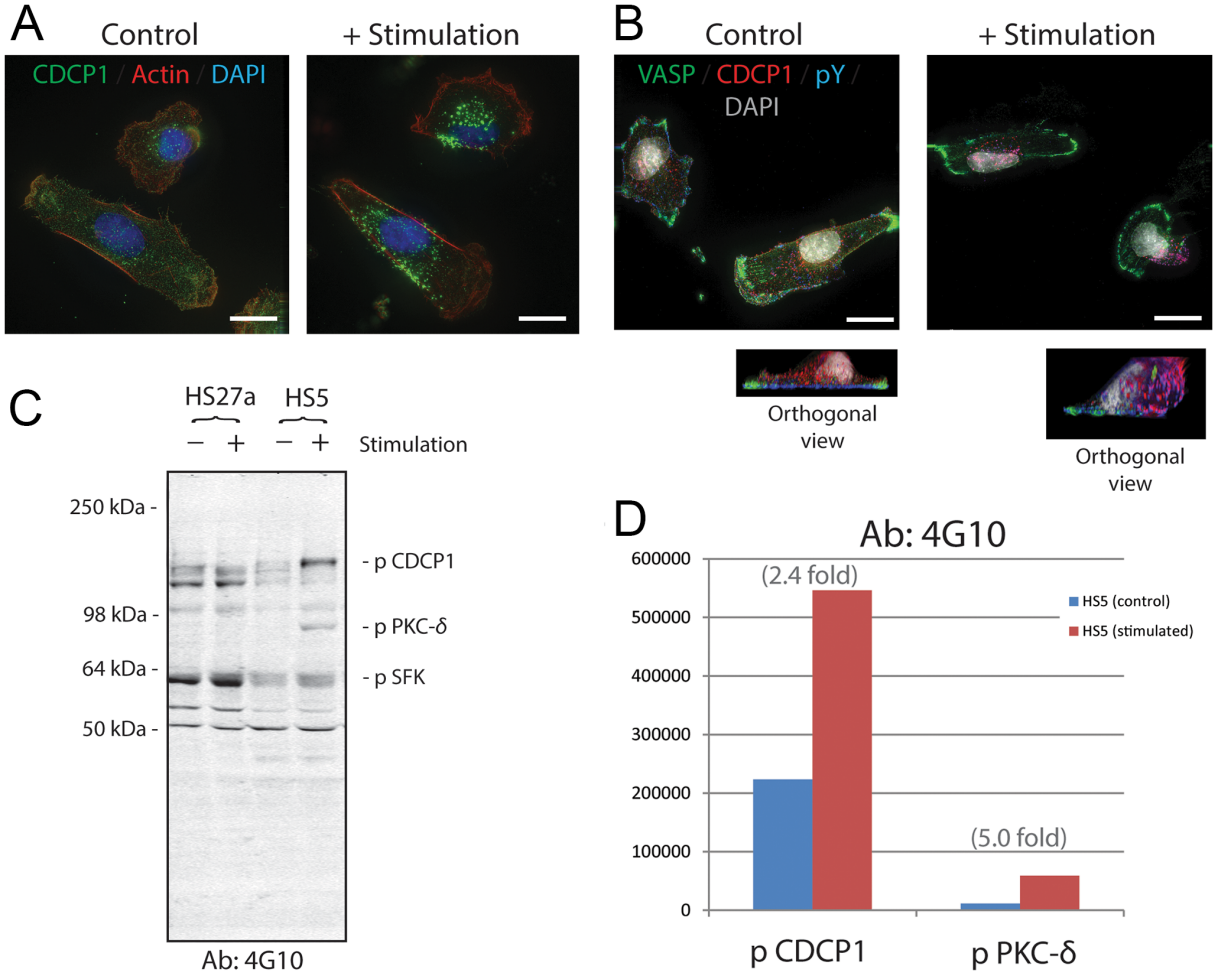
Immunofluorescence staining and protein blot analysis of CDCP1 in HS5 stromal cells before and after stimulation. **Panel A**: HS5 cells were treated with and without P3D9 stimulating antibody against CDCP1 for 30 minutes (right and left panels, respectively). They were then fixed and stained for CDCP1 (green), Actin (red) and Nuclei (DAPI, blue). Scale bars, 20 µm. **Panel B**: Control and stimulated HS5 cells were fixed and stained for VASP (green), CDCP1 (red), phosphotyrosine (pY, blue; detected by 4G10 antibody) and nuclei (DAPI, gray). Orthogonal images of the control and stimulated cells are shown. Pink fluorescence indicates the co-localization of CDCP1 and pY, suggesting that CDCP1 was tyrosine-phosphorylated. Original objective, X60. Scale bars, 20 µm. **Panel C**: Western blot analysis of detergent extracts of HS27a and HS5 cells before (−) and after (+) P3D9 stimulation. 4G10 antibody against pY was used. The bands of phosphorylated CDCP1, pSFK and PKC-δ were indicated according to the published studies [25], [46]. Identification of the PKC-δ band was confirmed by knock-down experiments of HS5 cells using siRNA for PKC-δ (Figure S3 in File S1). **Panel D**: Integrated pixel intensity of the bands of phosphorylated CDCP1 and PKC-δ in the blot of Panel C was quantitated by using Odyssey application software. Blue and red bars indicate HS5 cells before and after stimulation, respectively.

Two of the authors (WGC and EAW) established a panel of monoclonal antibodies against CDCP1, and one of them (P3D9) was found to activate CDCP1 [24], [25]. This agonistic antibody was used to elucidate the downstream signaling mechanisms in HS5 cells. In the steady state, CDCP1 in HS5 stromal cells is distributed evenly over the entire cell (Figure 2A and B, left panels). When the cells were cultured in the presence of antibody P3D9 for 5–30 minutes, a series of dynamic changes occurred: CDCP1 molecules aggregated and moved to the tip of the apical surface (Figure 2A and B, right panels). In contrast, vasodilator-stimulated phosphoprotein (VASP), a cell adhesion molecule at the site of focal adhesion associated with filamentous actin and regulated by PKA and PKC, did not show either aggregation or shifting to the tip of the apical side (green fluorescence in Figure 2B).

CDCP1 has a single intracellular domain with 5 tyrosine residues. Western blot analysis using 4G10 anti-phosphotyrosine (pY) antibody shows that phosphorylation of tyrosine residues in CDCP1 was upregulated at 2.4 fold after P3D9 stimulation (Figure 2C and D). Immunofluorescence staining shows that CDCP1 on the apical surface was not phosphorylated in the steady state (Figure 2B, left panel, red fluorescence). Co-localization of antibodies against CDCP1 and pY (4G10) on the apical side (Figure 2B, right panel, pink fluorescence) suggests that CDCP1 was tyrosine-phosphorylated and/or tightly associated with a tyrosine-phosphorylated protein upon stimulation with antibody P3D9.

To test if CDCP1 was directly phosphorylated, protein blot experiments were conducted. Western blot of the detergent extracts using 4G10 anti pY antibody shows that CDCP1 is a weakly tyrosine-phosphorylated protein at the steady state of HS5 cells (Figure 2C, – stimulation of HS5). After stimulation with antibody P3D9, phosphorylated CDCP1 and PKC-δ were quantitatively increased (Figure 2C and D, + stimulation of HS5). Identity of the PKC-δ band on the pY blot was confirmed by using HS5 cells after transfecting with siRNA for PKC-δ (Figure S3 in File S1, lanes 5 and 6). PKC-δ was not phosphorylated after stimulation in CDCP1-knocked down cells (Figure S3 in File S1, lane 4), suggesting that phosphorylation of PKC-δ is a downstream event after the CDCP1 stimulation. As expected, phosphorylated CDCP1 and phosphorylated PKC-δ are completely absent in HS27a cells (Figure 2C, left two lanes).

**Figure 3:**
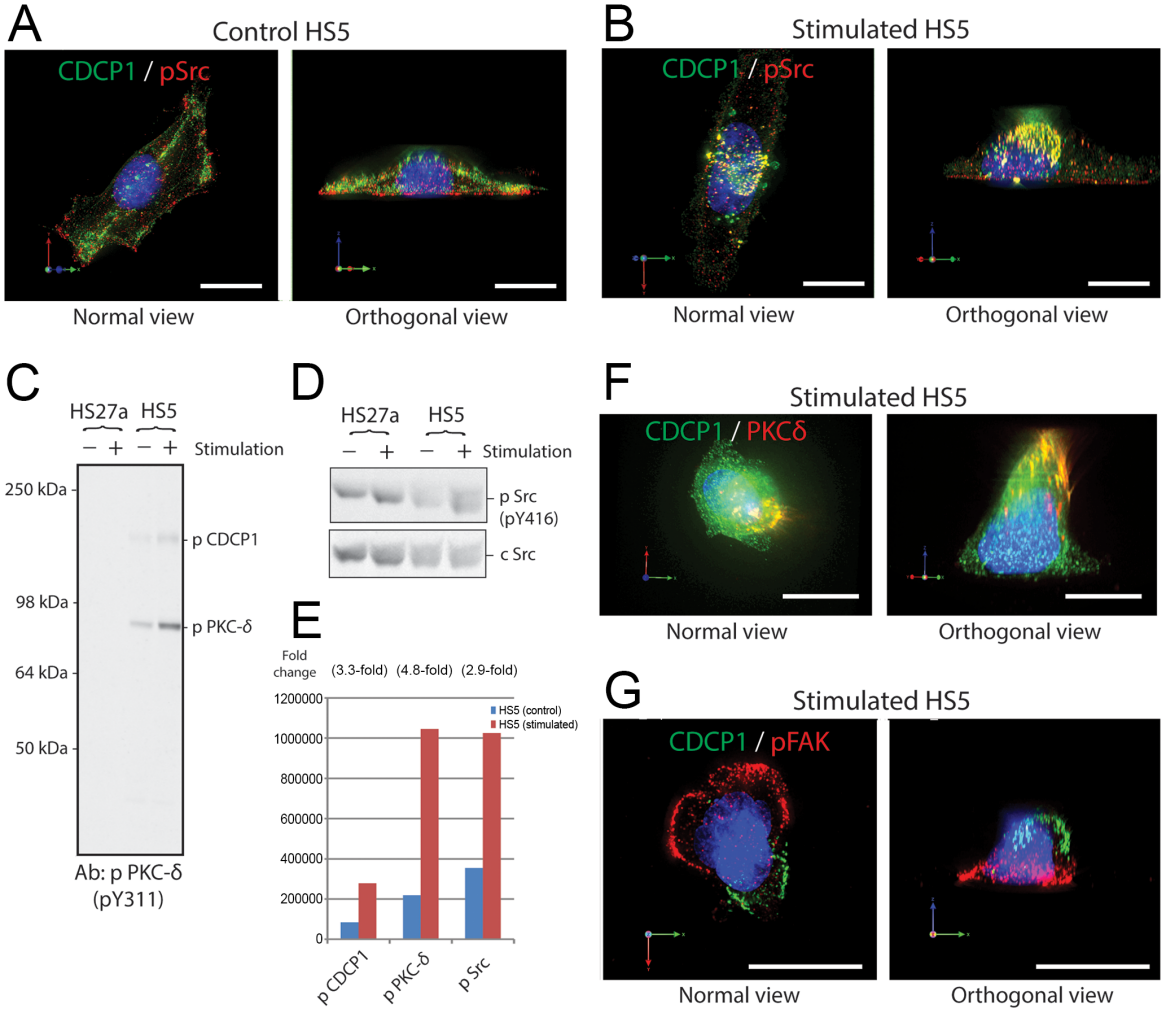
Co-localization of phosphorylated Src and PKC-δ with CDCP1 after activation in HS5 stromal cells. Immunofluorescence staining was conducted in control HS5 cells (panel A) and the cells after 30 minutes of P3D9 stimulation (panels B, F and G) were fixed and stained for CDCP1 (green), nuclei (blue), and one of the signaling molecules (red) such as phosphorylated SFK (pSrc in panels A and B), PKC-δ (panel F) and phosphorylated FAK (pFAK in panel G). Images of normal and orthogonal views were shown in each staining. Western blots using antibodies against phosphorylated PKC-δ, phosphorylated SFK (pSrc), and cellular Src (cSrc), and the quantitation of the band intensity were shown in panels C-E. **Panels A and B**: CDCP1 (green) were widespread on both apical and basal surfaces of control HS5 cell, and pSrc mostly localized in the basal side of the cells (panel A). In contrast, CDCP1 shifted toward the apical tip of the cells after stimulation (panel B). CDCP1 associated with pSrc, indicated by yellow fluorescence. In the orthogonal view, red fluorescence was observed on the basal side of the cell, suggesting there were some pSrc left unassociated with CDCP1 on the basal side of the cells. Original objective, X60. Scale bars, 20 µm. **Panel C**: Western blot analysis of detergent extracts of HS27a and HS5 cells using anti-phosphorylated PKC-δ antibodies (pY311). CDCP1 and PKC-δ share the same epitope for pY311 antibodies. **Panel D**: Western blot analysis of detergent extracts of HS27a and HS5 cells using antibodies against phosphorylated SFK-pY416 (upper panel) and cellular Src as a loading control (lower panel). **Panel E**: Integrated pixel intensity of the bands of phosphorylated CDCP1, PKC-δ and SFK in the blot of panels C and D was quantitated by using Odyssey application software. Blue and red bars indicate HS5 cells before and after stimulation, respectively. **Panel F**: Localization of CDCP1 (green) and PKC-δ is shown in the cells after stimulation. Most PKC-δ was associated with CDCP1 (yellow color) and moved to the apical tip of the cells. No PKC-δ was left in the basal side of the cells, indicated by the lack of the red fluorescence. Original objective, X60. Scale bars, 20 µm. **Panel G**: Stimulated cells were stained for CDCP1 (green) and pFAK (red). In contrast to pSFK and PKC-δ, pFAK was not associated with CDCP1 in the stimulated cells and remained on the basal side of the cells. Original objective, X60. Scale bars, 20 µm.

CDCP1 was reported to be phosphorylated by Src-family protein kinases (SFKs) and to activate SFKs in metastasizing melanoma and tumor cell lines [24], [35]–[37]. Distribution of phosphorylated SFKs (pSFKs) in control and stimulated HS5 cells was examined by immunofluorescent microscopy (Figure 3A and B, respectively). Although CDCP1 and pSFK distributed widely throughout the control cells, pSFK bound to CDCP1 and moved toward the apical surface of the cells after stimulation (yellow fluorescence in the apical side in the right panel of Figure 3B). Some pSrc remained on the basal side and unattached to CDCP1 after stimulation (red fluorescence in the basal side in the right panel of Figure 3B). Western blot analysis shows a 2.9-fold increase in pSFK in HS5 cells after P3D9 stimulation (Figure 3D and E). HS27a cells did not change levels of pSFK after stimulation (Figure 3D).

In skin epidermal cells and epithelial tumor cells, PKC-δ is known to bind to CDCP1 after stimulation [25], [38]–[40]. Western blot analysis using Y311 anti-phosphorylated PKC-δ antibodies indicates a 4.8-fold increase in phosphorylated PKC-δ in HS5 cells after P3D9 stimulation (Figure 3C and E). This correlates well with the levels of phosphorylated PKC-δ in the blot using 4G10 anti-pY antibody (5.0 fold after stimulation shown in Figure 2D). Figure 3F shows the distribution of PKC-δ in HS5 cells after stimulation by immunofluorescence staining. Most of PKC-δ bound to CDCP1 and moved to the apical side. There were only a few PKC-δ molecules left bound to CDCP1 in the basal membrane, confirmed by using the total internal reflection fluorescence microscopy (TIRF) (Figure S4 in File S1). In contrast, phosphorylated focal adhesion kinase (pFAK) did not bind to or shift with CDCP1 after stimulation (Figure 3G). These data suggest that the downstream signaling pathway of the stimulated CDCP1 is preferential for pSFK and PKC-δ in HS5 cells.

**Figure 4:**
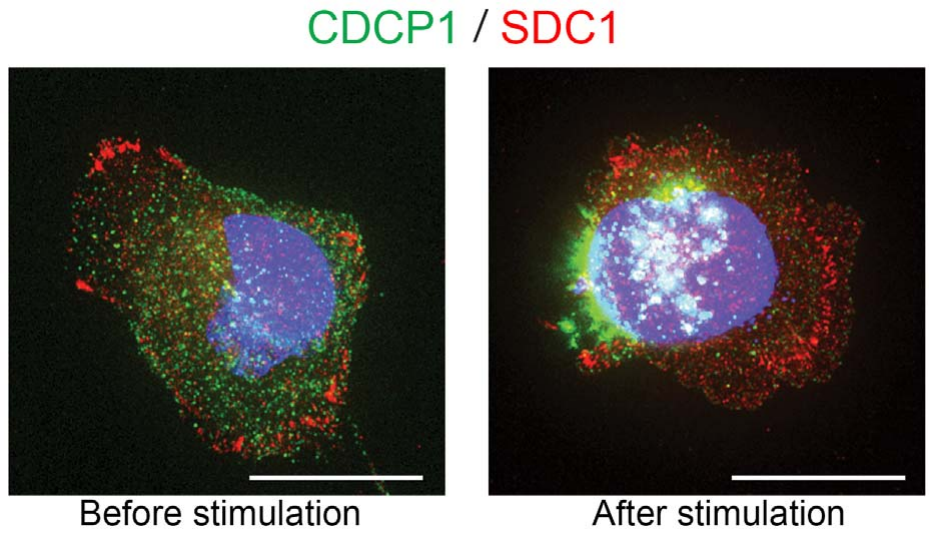
An adhesion molecule, Syndecan-1 (SDC1), was not associated with CDCP1 and did not respond to the stimulation of CDCP1. HS5 cells were fixed before and after stimulation, and stained for CDCP1 (green), SDC1 (red) and nuclei (blue). SDC1 localized to the basal surface of the cells, and were especially concentrated in the focal adhesion areas in both control and stimulated cells. CDCP1 shifted toward the tip of the cells after stimulation, whereas SDC1 did not. White staining indicates CDCP1 localizing over the nucleus. Original objective, X60. Scale bars, 20 µm.

Syndecan-1 (SDC1)/CD138 is an adhesion molecule expressed in the area of focal adhesion. It has been proposed that CDCP1 interacts with SDC1 in a human breast cancer cell line, MDA-468 [35]. Since HS5 stromal cells express both CDCP1 and SDC1, we tested if CDCP1 and SDC1 bind in steady and stimulated states by immunofluorescence staining (Figure 4). We found that SDC1 was enriched in focal adhesion in both steady and stimulated states, whereas CDCP1 showed dynamic change after stimulation as consistently shown in Figures 2–3. Co-localization analysis using softWoRx software confirmed that CDCP1 and SDC1 did not co-localize on basal or apical surface in unstimulated HS5 cells (Figure S5 in File S1).

### CDCP1+ stromal cells are present in bone marrow

**Figure 5:**
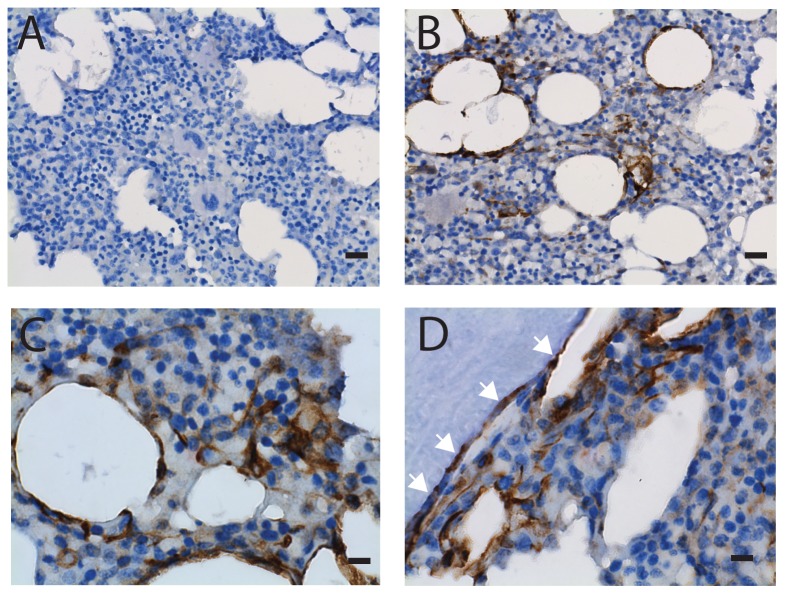
Immunohistochemical detection of CDCP1+ cells in marrow biopsy. Marrow biopsies of the healthy donors were subjected to immunohistochemistry using isotype-matched control antibody (panel A) and an anti-CDCP1 antibody (Cell Signaling Technology, panels B-D). Positive cells were stained in brown and nuclei were counter-stained using hematoxylin (blue). Panels B and C show CDCP1+ cells clustered near some fat cells. Panel D shows CDCP1+ cells near bone. White arrows indicate that the thin-flattened cells surrounding bone were CDCP1-positive. Original objective, X40 for panels A and B (black bars indicate 20 µm); X100 for panels C and D (black bars indicate 8 µm).

CDCP1+ cells were detected in bone marrow biopsies by immunohistochemistry (Figure 5). Specific staining was validated using specimens of human tonsil (Figure S6 in File S1) as positive controls. Clusters of CDCP1+ cells were found in marrow near fat cells and surrounding bone. Sinusoids and capillaries in bone marrow, where CD146+ cells reside, were negative for CDCP1. Dual immunohistochemistry using antibodies against CDCP1 and CD146 was conducted, and confirmed that CDCP1+ and CD146+ cells were present in different locations (Figure S7 in File S1). These data show that CDCP1+ cells are present in marrow microenvironment and do not co-localize with the CD146+ stromal cells.

### Knock down of CDCP1 by siRNA upregulates cytokine expression

**Figure 6:**
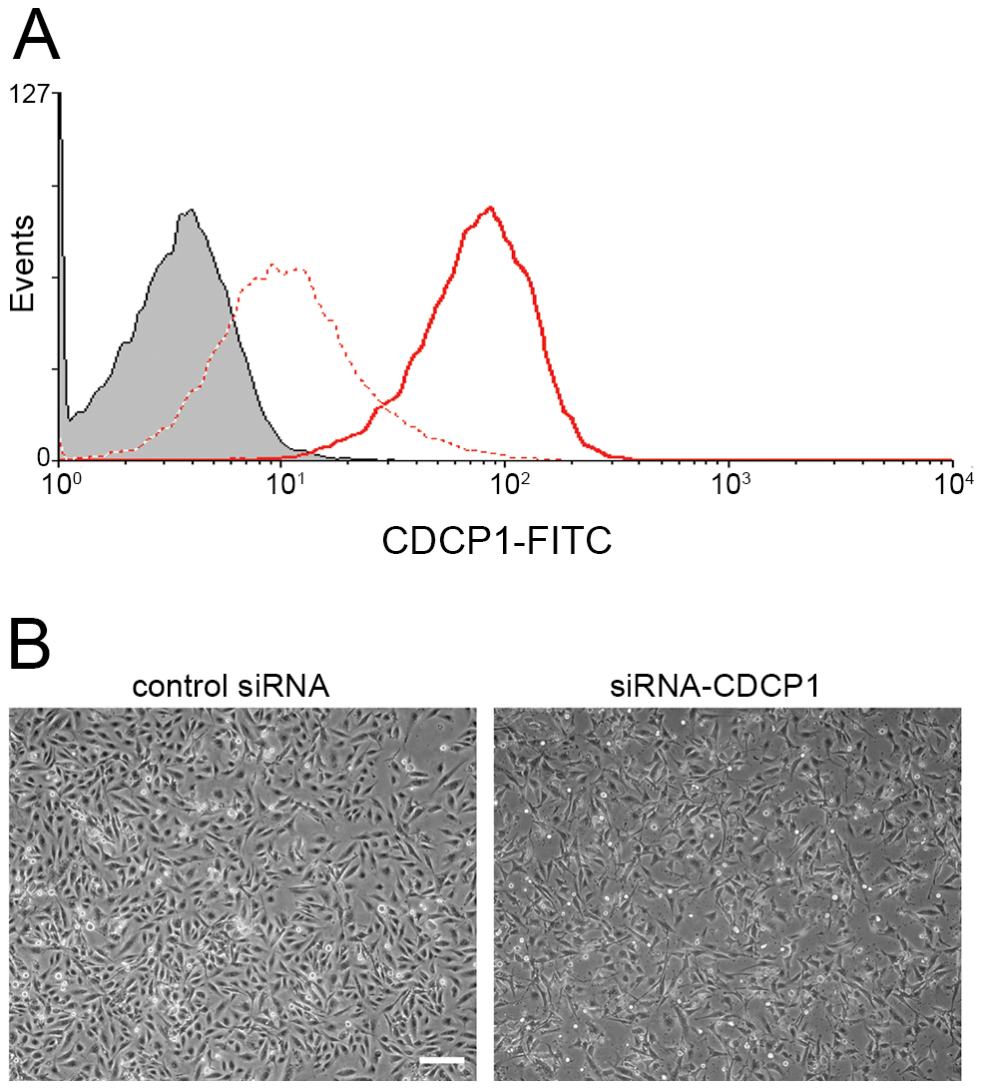
Knock-down of CDCP1 in HS5 cells by siRNA. **Panel A**: Surface protein expression of CDCP1 was analyzed by flow cytometry. HS5 cells were transfected with siRNA for CDCP1 (dotted red line) or GFP (solid red line) and cultured for 3 days, followed by flow cytometry for CDCP1. Gray area indicates the isotype-matched control. Expression of an unrelated protein, Integrin alpha 3 (ITGA3/CD49c) did not change (shown in Figure S8 in File S1). **Panel B**: Phase contrast images of the transfected cells are shown. HS5 cells were transfected with siRNA for GFP (left panel) or CDCP1 (right panel) and cultured for 3 days. Original objective, X20. A white bar indicates 200 µm.

Next, we tried to elucidate the function of CDCP1 in the stromal cells by knocking down (KD) CDCP1 expression using siRNA technology. Four siRNAs were tested to identify the siRNA that provided optimal reduction in CDCP1 expression, with minimal obvious off-target effects (Figure S8 in File S1). After transfecting HS5 cells with siRNA for CDCP1, surface expression of CDCP1 transiently decreased to 10% of control levels from 2 to 3 days post transfection (Figure 6A). Cell growth was slightly retarded, and the cells had elongated cell extensions after knock down (Figure 6B).

**Figure 7:**
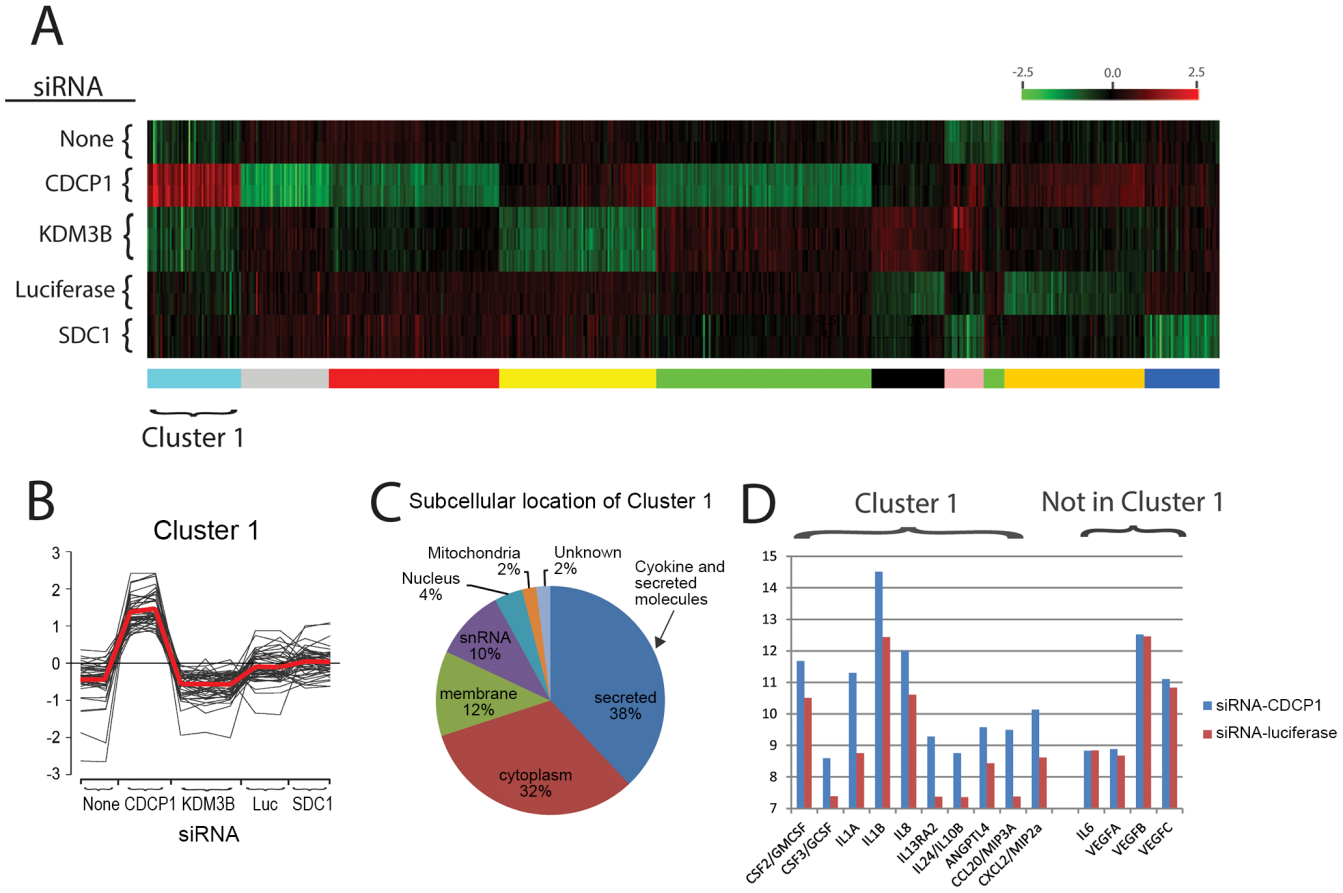
K-means clustering analysis of microarray data obtained HS5 cells after knocked down for CDCP1 and unrelated control gene targets. Normalized log2 intensities were probe-wise median centered across all conditions. **Panel A**: A heat map of clusters generated from the 573 probes showing differential gene expression (defined as |log_2_(ratio)| ≥1 and FDR of 5%) in the comparison between control and knock downed HS5 cells. **Panel B**: Log-ratio values for the 50 probes comprised of Cluster 1 which show upregulation in CDCP1 KD cells. Red line is the mean expression value of the cluster members. **Panel C**: A pie chart showing subcellular location of probes in Cluster 1. Blue pie indicates cytokine and secreted molecules. **Panel D**: Log 2 gene expression values of cytokines and chemokines present or absent in Cluster 1. Blue and red bars indicate HS5 cells transfected with siRNA for CDCP1 or luciferase, respectively.

Global gene expression profiling using microarray technology was used to elucidate CDCP1 KD-associated changes in gene expression of HS5 cells (Figure 7). siRNA for unrelated gene targets (SDC1, lysine-specific demethylase (KDM3B), and luciferase (Luc)) were used as controls. Heat map and clustering analysis reveals 50 gene probes uniquely upregulated in CDCP1-KD cells (Cluster 1 in Figure 7A and B). Almost 40% of the gene probes in Cluster 1 are secreted molecules such as cytokines and chemokines (Figure 7C). Figure 7D shows gene expression of some cytokines present or absent in Cluster 1. The gene expression files are available in the Gene Expression Omnibus series accession GSE53199 (http://www.ncbi.nlm.nih.gov/geo/query/acc.cgi?acc=GSE53199).

**Figure 8:**
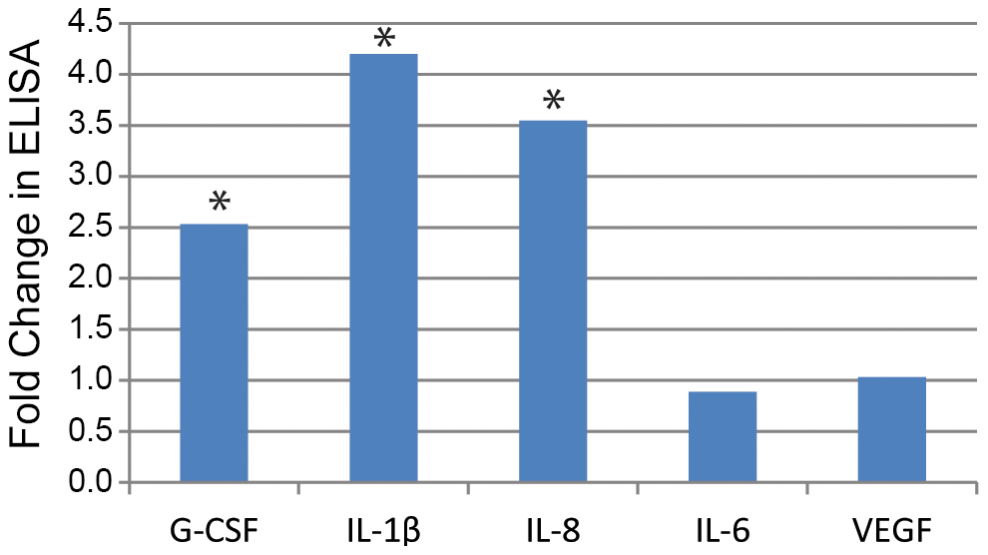
Cytokine secretion of HS5 stromal cells. HS5 cells were transfected with siRNA for CDCP1 or luciferase (control). After feeding fresh media at 1 day post transfection, the cells were cultured for 48 hours, and cell supernatants were collected. The cytokine levels were measured by ELISA (n = 4). Y axis indicates fold changes in the cells which were infected with siRNA for CDCP1 compared to the control siRNA. Asterisks indicate significant difference (p = 0.0003∼0.001).

Next, we tested if the knock down of CDCP1 will result in changes in protein expression and secretion of the cytokines and chemokines by ELISA (Figure 8 and Table 1). As suggested by gene expression profiling, secretion of G-CSF, GM-CSF, IL-1a, IL-1b and IL-8 were significantly upregulated in the knocked down-cells, whereas the expression of IL-6 and VEGF did not change. Lastly, we determined if PKC-δ, a downstream effector for CDCP1 in HS5 cells (Figure 3), contributed to the production of cytokines. Interestingly, knockdown of PKC-δ failed to change levels of secreted GCSF (1.1-fold for PKC-δ-KD versus 2.4-fold for CDCP1-KD) or IL-8 (1.7-fold for PKC-δ-KD versus 8.4-fold for CDCP1-KD) in HS5 cells, suggesting that a novel signaling mechanism may link CDCP1 to regulation of cytokines.

**Table 1:** Cytokine secretion from HS5 cells after transfecting with siRNA for CDCP1 or Luciferase (control).

	siRNA-CDCP1 (pg/mL)	Control siRNA (pg/mL)	Fold	*P* value
**G-CSF**	15050+/−1626	5950+/−1904	2.5	0.003
**GM-SF**	1553+/−178	1205+/−114	1.3	0.04
**IL-1a**	145+/−10	97+/−4	1.5	0.007
**IL-1b**	97+/−11	23+/−1	4.2	0.0003
**IL-8**	34539+/−5388	9741+/−2593	3.5	0.001
**IL-6**	2203+/−244	2478+/−134	0.9	N.S.
**VEGF**	1905+/−191	1846+/−158	1.0	N.S.

## Discussion

Primary marrow stromal cell cultures contain a heterogeneous population of cells that differ in morphology, immunophenotype [41], [42], and function. This heterogeneity is further complicated by the fact that primary stromal cell cultures can retain, through several passages, various amounts of hematopoietic cells, predominantly monocytes and macrophages [43]. To better understand the functional heterogeneity of stromal cell cultures we isolated and immortalized distinct mesenchymal stromal cell clones, HS5 and HS27a [21], which are fibroblasts and as shown in this report can be easily distinguished by expression of CDCP1 and CD146 respectively. HS5 stromal cells secrete copious amounts of multiple cytokines and chemokines that support myeloid differentiation and expansion [21]. This characteristic is very unique to CDCP1+ HS5 cells but not to CDCP1–/CD146+ HS27a cells or the majority of primary marrow stromal cells. The present data suggest that CDCP1, in addition to being a reliable marker for a functionally distinct subset of marrow fibroblasts, may also play a role in modulating the production of myelopoietic cytokines secreted by this subset of cells.

CDCP1 is a glycoprotein with a single transmembrane domain, 3 extracellular CUB domains and a large intracellular domain with 5 tyrosine residues. CDCP1 is reportedly expressed in normal epithelial cells, and overexpressed in epithelial tumor cells such as colon, breast, lung, renal and pancreatic cancers [44]–[46]; expression of CDCP1 by bone marrow stromal cells has not been previously reported. Immunohistochemistry detection of CDCP1 in marrow biopsies in this study shows clusters of positive cells located; some next to bone, some circumscribing areas of fat, others surrounded by active hematopoiesis. Unlike the CD146 positive cells, CDCP1+ stromal cells were not detected in peri-sinusoidal locations.

Previously the distribution of CD146- and CD146+ subsets of marrow stromal cells was shown to be correlated with donor age [47], [48]. The current study neither supports nor contradicts this finding. Although the youngest donor in the current study (Donor #1) has the highest percentage of CD146-negative stromal cells (Figure 1E), the remaining 4 donors vary in age from 40 to 54 years, which does not constitute a sufficient range or sample size for statistics. Furthermore, in the previous study both CD146- and CD146+ cells expressed CD271/NGFR, whereas in the studies reported here CDCP1+/CD146- cells do not express CD271, suggesting the CDCP1 positive cells may represent a subset of CD146- cells.

Flow cytometry data in Figure 1E show CD146 and CDCP1 expression by in vitro expanded stromal cells. Both populations, CD146+ and CDCP1+, could be detected in all tested marrow samples, freshly isolated or expanded, albeit in widely varying proportions. At this time we speculate that this may be attributable to the spatially separated, histologically distinct areas that are evident in marrow tissue in large mammals. In agreement with this, the results in this report show the CDCP1+ and CD146+ cell populations are concentrated in different locations in bone marrow. Whether these two fibroblast cell types with opposing but complementary hematopoietic regulatory functions occur in a specific relative frequency in health and disease remains to be determined. The development of antibodies that distinguish the two makes it possible to address this issue.

Following stimulation of epithelial cells, CDCP1 is known to be phosphorylated on the tyrosine residues in the intracellular domain [25], [49]. The phosphorylation of CDCP1 occurs in a SFK-dependent manner, and is followed by association with PKC-δ. Our data show that CDCP1 in marrow stromal cells is also phosphorylated after stimulation by antibody binding, and binds to pSFKs. The activated PKC-δ-bound CDCP1 then moves to the apical surface, and the cells go through morphological changes. These changes occur within 30 minutes of stimulation. These data suggest that CDCP1 expressed on the surface of marrow stromal cells is active and responds after receiving the outside-in signals.

Outcomes beyond CDCP1 participation in the phosphorylation by SFK and the regulation of PKC-δ is suggested by metastasis and survival of tumor cells. Here, using an RNAi-based approach against CDCP1, we found that knock-down of stromal CDCP1 increased expression and secretion of a subset of cytokines including G-CSF, GM-CSF and IL-8, but not IL-6 or VEGF. These cytokines from HS5 are active and can expand hematopoietic progenitor cells in vitro [21]. Skin epithelial cells and other CDCP1+ epithelial cell lines secrete IL-8 mediated by PKC-δ [50], [51]. Knock-down of CDCP1 by siRNA in these epithelial cells does not upregulate IL-8 expression (unpublished observation by WGC). Therefore, the association of IL-8 upregulation with the knock-down of CDCP1 is unique to marrow stromal cells. Also of interest is the disparate response between IL-8 (increased) and IL-6 (decreased) expression seen in association with CDCP1 knock-down, since these two cytokines are often co-regulated. These data suggest a potential mechanism whereby the levels of CDCP1 expressed by a population of marrow fibroblasts may regulate cytokine production in those cells. Exactly how CDCP1 levels can be modulated in vivo is not known. Therefore, developing models which can examine this potential regulatory element will add to our appreciation of the complexity of hematopoietic regulation and may lead to new therapeutic targets for marrow dysplasia and fibrotic diseases. The ability to identify and isolate CDCP1 positive cells increases the possibility of such a model.

## Supporting Information

File S1 Contains the following files: **Table S1**. List of antibodies. **Figure S1**. Surface protein expression of CDCP1 in the stromal cell. **Figure S2**. Gene expression of CDCP1 in cytokine-stimulated stromal cells. **Figure S3**. Identification of phosphorylated PKC-δ by knocking down HS5 cells with siRNA for PKC-δ. **Figure S4**. Visualization of CDCP1 and PKC-δ on the basal surface of HS5 cells by the total internal reflection fluorescence (TIRF) microscopy. **Figure S5**. Dual staining of CDCP1 and SDC1 in HS5 cells. **Figure S6**. Validation of immunohistochemistry of CDCP1. **Figure S7**. Dual immunohistochemistry of CDCP1 and CD146. **Figure S8**. Knock-down of CDCP1 by various siRNAs.
(DOCX)
