# Supplementary material for: CDCP1 Identifies a CD146 Negative Subset of Marrow Fibroblasts Involved with Cytokine Production
Source: PLoS One. 2014 Oct 2;9(10):e109304. doi: 10.1371/journal.pone.0109304 (PMC4183599; doi:10.1371/journal.pone.0109304)
Supplement: File S1 — Contains the following files: Table S1. List of antibodies. Figure S1. Surface protein expression of CDCP1 in the stromal cell. Figure S2. Gene expression of CDCP1 in cytokine-stimulated stromal cells. Figure S3. Identification of phosphorylated PKC-δ by knocking down HS5 cells with siRNA for PKC-δ. Figure S4. Visualization of CDCP1 and PKC-δ on the basal surface of HS5 cells by the total internal reflection fluorescence (TIRF) microscopy. Figure S5. Dual staining of CDCP1 and SDC1 in HS5 cells. Figure S6. Validation of immunohistochemistry of CDCP1. Figure S7. Dual immunohistochemistry of CDCP1 and CD146. Figure S8. Knock-down of CDCP1 by various siRNAs. (DOCX) [file pone.0109304.s001.docx]

**SUPPORTING INFORMATION (ONLINE ONLY)**

**FOR**

**CDCP1 identifies a CD146 negative subset of marrow fibroblasts involved with cytokine production**

Mineo Iwata^1^, Beverly Torok-Storb^1^, Elizabeth A. Wayner^1^, and William G. Carter^1^

^1^Fred Hutchinson Cancer Research Center, Seattle, WA, USA.

**Supporting Information Legends**

**Supporting Table S1 in File T1.** List of antibodies

**Supporting Figure S1 in File S1.** Surface protein expression of CDCP1 in the stromal cell

**Supporting Figure S2 in File S2.** Gene expression of CDCP1 in cytokine-stimulated stromal cells

**Supporting Figure S3 in File S3.** Identification of phosphorylated PKC-δ by knocking down HS5 cells with siRNA for PKC-δ

**Supporting Figure S4 in File S4.** Visualization of CDCP1 and PKC-δ on the basal surface of HS5 cells by the total internal reflection fluorescence (TIRF) microscopy

**Supporting Figure S5 in File S5.** Dual staining of CDCP1 and SDC1 in HS5 cells

**Supporting Figure S6 in File S6.** Validation of immunohistochemistry of CDCP1

**Supporting Figure S7 in File S7.** Dual immunohistochemistry of CDCP1 and CD146.

**Supporting Figure S8 in File S8.** Knock-down of CDCP1 by various siRNAs.

**Supporting Table S1. List of antibodies**

| **Antibody** | **Company** | **Catalogue** | **Clone/Reagent** | **Isotype** |
| --- | --- | --- | --- | --- |
| Actin | Life Technologies | A12381 | Phalloidin-Alexa Fluor 594 | N/A |
| CDCP1 | in house | N/A | P3D9 | mouse IgG1 |
| CDCP1 | in house | N/A | P1C3 | mouse IgG1 |
| CDCP1 | in house | N/A | P5H10 | mouse IgG1 |
| CDCP1-FITC | BioLegend | 324003 | CUB1 | mouse IgG1 |
| CDCP1 | Cell Signaling Technologies | 4115 | N/A | rabbit polyclonal |
| pFAK (pY397) | Biosource | 44-624G | N/A | rabbit polyclonal |
| PKC-δ | Santa Cruz Biotechnology | sc-937 | C-20 | rabbit polyclonal |
| pPKC-δ | Cell Signaling Technologies | 2055 | Y311 | rabbit polyclonal |
| cSrc | Santa Cruz Biotechnology | sc-18 | N/A | rabbit polyclonal |
| pSrc (pY416) | Cell Signaling Technologies | 2101 | N/A | rabbit polyclonal |
| pY | in house | N/A | 4G10 | mouse IgG2b |
| SDC1 | Sigma | HPA006185 | N/A | rabbit polyclonal |
| VASP | BD Transduction Laboratories | 610447 | 43/VASP | mouse IgG1 |

**Supporting Figures**


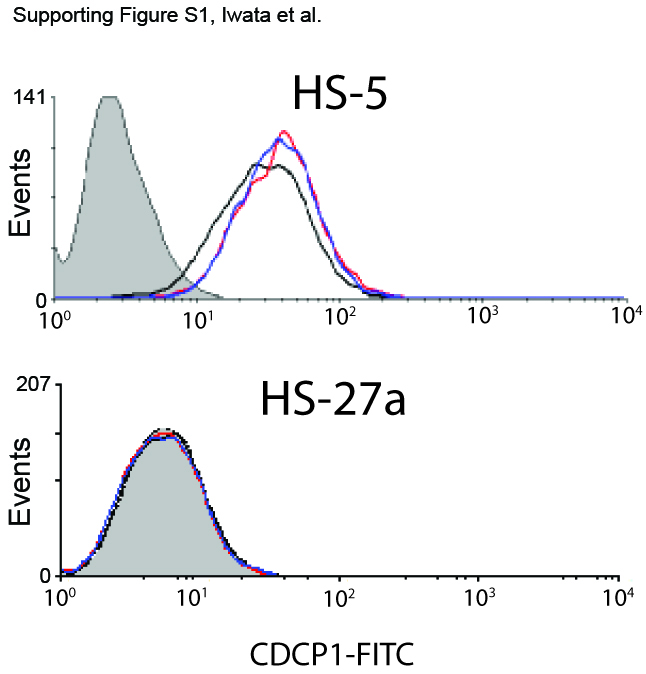


**Supporting Figure S1. Surface protein expression of CDCP1 in the stromal cells.**

Protein expression of CDCP1 on the surface of HS5 (upper panel) and HS27a (lower panel) was determined by flow cytometry using P1C3 (blue line), P3D9 (red line) and P5H10 (black line) antibodies against CDCP1. These monoclonal antibodies recognize different epitopes on CDCP1. These data show that all three epitopes are expressed on the cellular surface of HS5. Gray area shows the isotype-matched control.


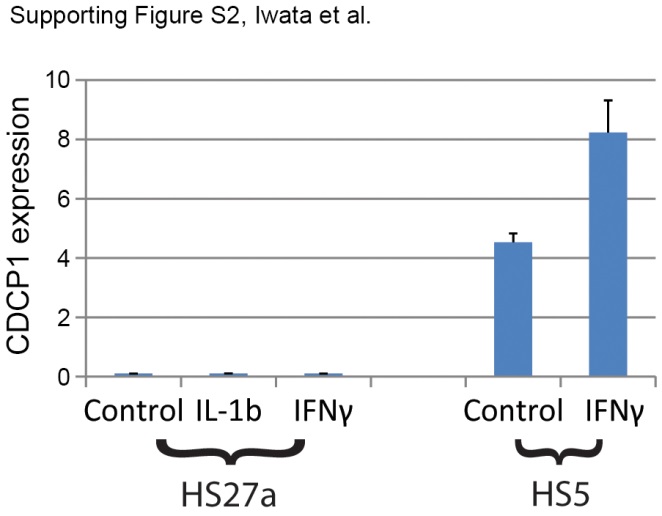


**Supporting Figure S2. Gene expression of CDCP1 in cytokine-stimulated stromal cells.** CDCP1- HS27a and CDCP1+ HS5 stromal cells were cultured in the presence of 2 ng/mL of IL‑1β (4 hours or 3 days) or 1 ng/mL of IFNγ (24 hours). After IL-1b or IFNγ stimulation, the cells were harvested and total RNA was isolated. Levels of CDCP1 transcripts were determined by quantitative PCR after normalized to the house-keeping gene. CDCP1 expression in HS27a was not induced by the cytokine stimulation.


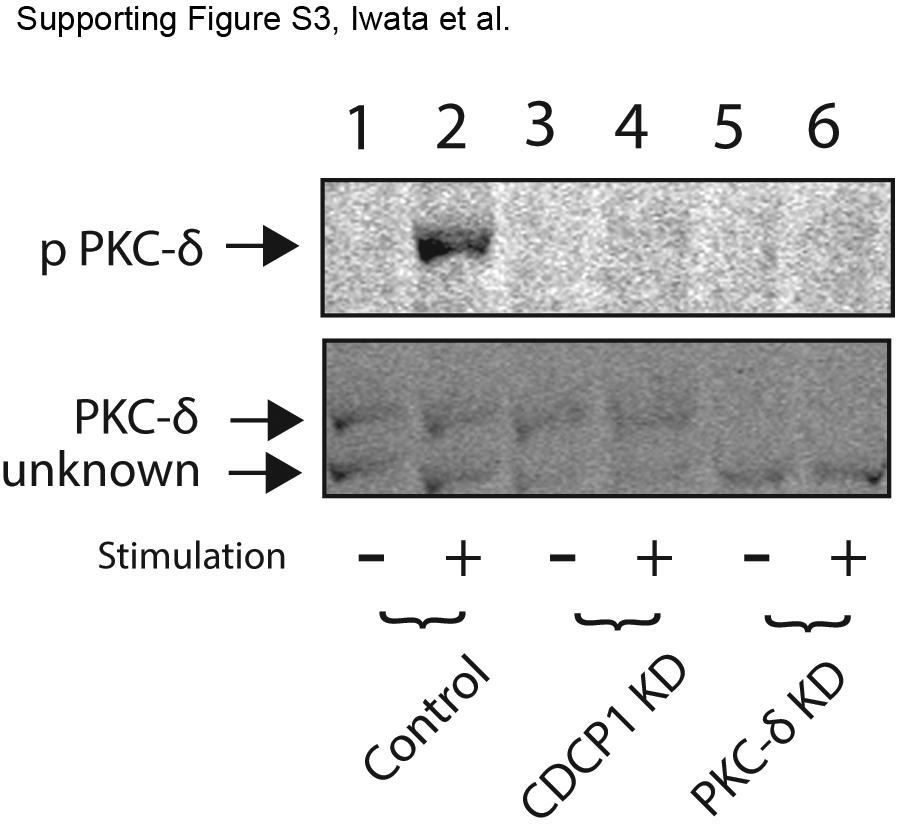


**Supporting Figure S3. Identification of phosphorylated PKC-δ by knocking-down HS5 cells with siRNA for PKC-δ.** HS5 cells were transfected with siRNAs for the negative control gene (lanes 1 and 2), CDCP1 (lanes 3 and 4) and PKC-δ (lanes 5 and 6). The cells were stimulated with (+) or without (-) P3D9 antibody for 15 minutes. A Western blot of the Triton-X-100 extracts was prepared. Sixty micrograms of proteins were loaded in each lane, and antibodies against PKC-δpY311 (upper panel) and PKC-δ (lower panel) were used. Phosphorylated and unphosphorylated PKC-δ bands were not detected in PKC-δ KD cells after stimulation (lane 6, upper and lower panels), confirming that the band of phosphorylated protein in the upper panel (lane 2) is PKC-δ. Phosphorylated PKC-δ was detected after stimulation in the control cells but not in CDCP1 KD cells (lanes 2 and 4, upper panel), suggesting that PKC-δ is a down-stream event of CDCP1 stimulation.


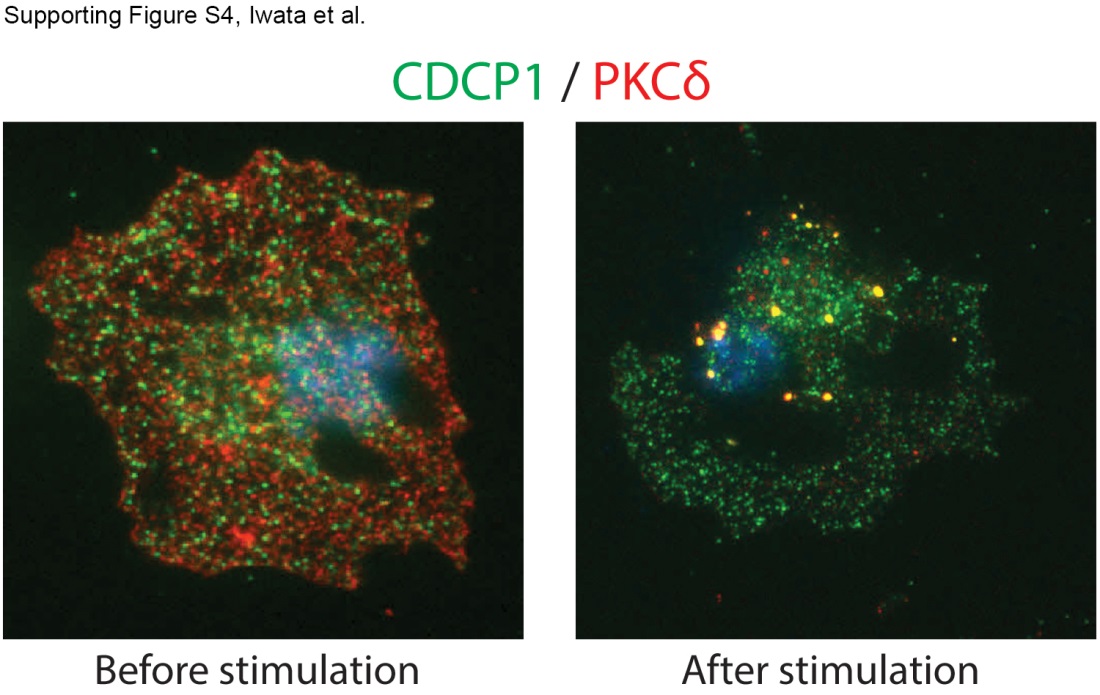


**Supporting Figure S4: Visualization of CDCP1 and PKC-δ on the basal surface of HS5 cells by the total internal reflection fluorescence (TIRF) microscopy.** HS5 cells before and after P3D9 stimulation were fixed and stained for CDCP1 (green), PKC-δ (red) and nuclei (DAPI, blue). CDCP1 and PKC-δ on the basal membrane were detected by TIRF microscopy. Before stimulation, CDCP1 and PKC-δ distributed evenly on the basal membrane, and were not bound together. In contrast, only a few PKC-δ molecules remain in the basal membrane after stimulation. The PKC-δ co-localized well to CDCP1, indicated by yellow fluorescence. Total internal reflection fluorescence microscope (Nikon Eclipse Ti) with a 100X/1.49 oil Apo TIRF lens (Nikon) and an iXON X3 camera (ANDOR Technology, Belfast UK) was used.


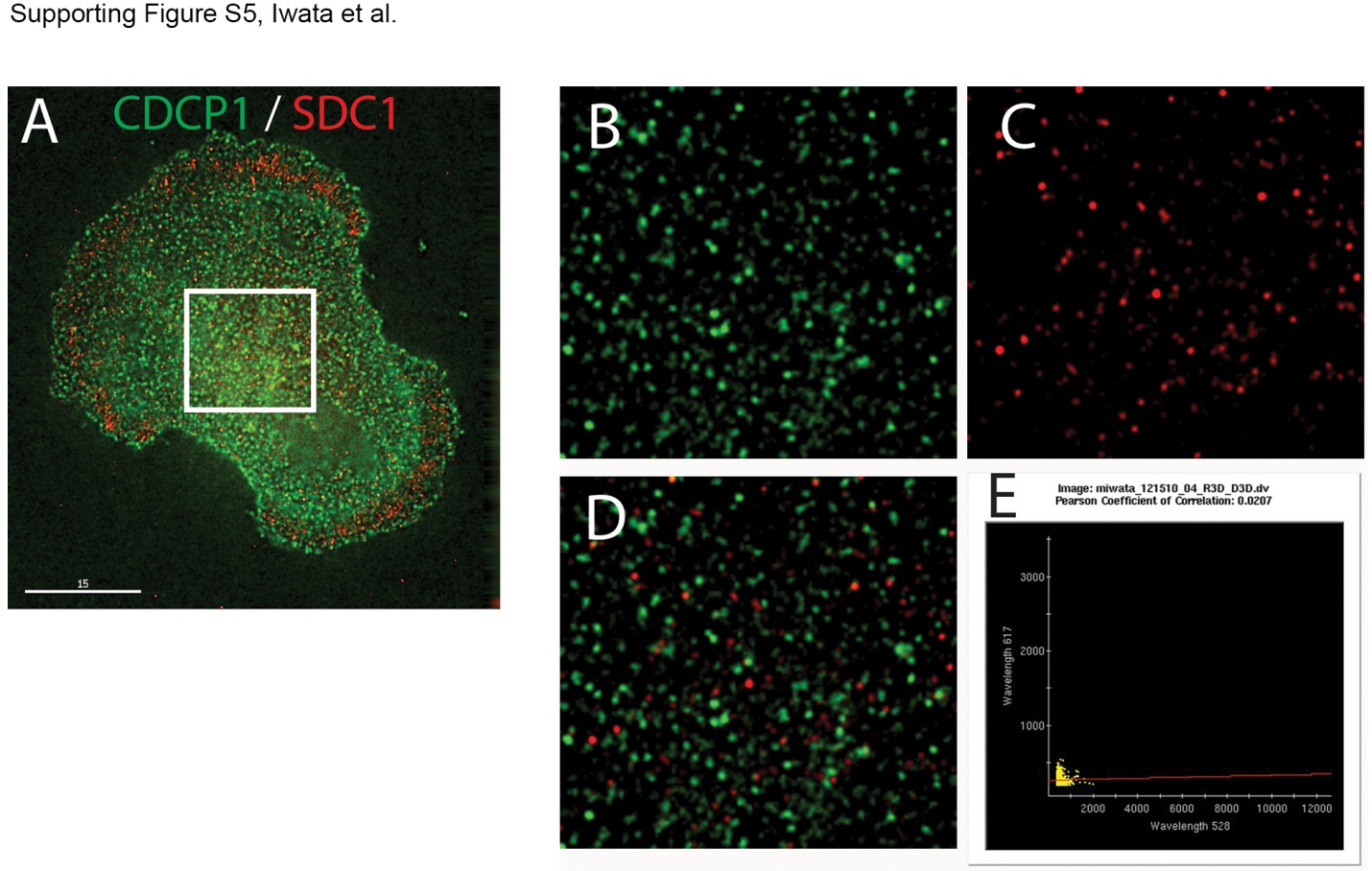


**Supporting Figure S5. Dual staining of CDCP1 and SDC1 in unstimulated HS5 cells.** The co-localized of CDCP1 and SDC1 was evaluated before treatment with P3D9 antibody. HS5 cells were dual-stained for CDCP1 (green) and SDC1 (red) in panel A. The boxed area was enlarged for fluorescence of CDCP1 (panel B), SDC1 (panel C) and combined (panel D). There was no co-localization of green and red signals. Panel E shows the co-localization analysis between the green and red channels performed on a representative area using softWoRx software (Applied Precision). X axis, green fluorescence intensity; Y axis, red fluorescence intensity.


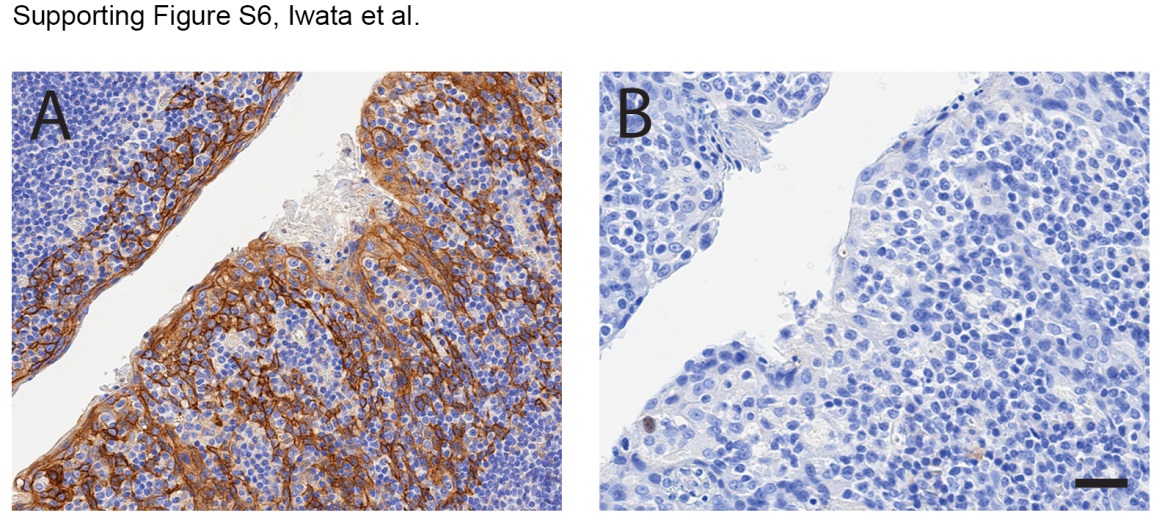


**Supporting Figure S6. Validation of immunohistochemistry of CDCP1.** Specimens of human tonsil were stained using anti-CDCP1 antibodies (panel A) or isotype-matched control (panel B). Brown staining is for CDCP1, and the nuclei were counter-stained by hematoxilin (blue). Original objective, X40. Scale bar, 50 μm.


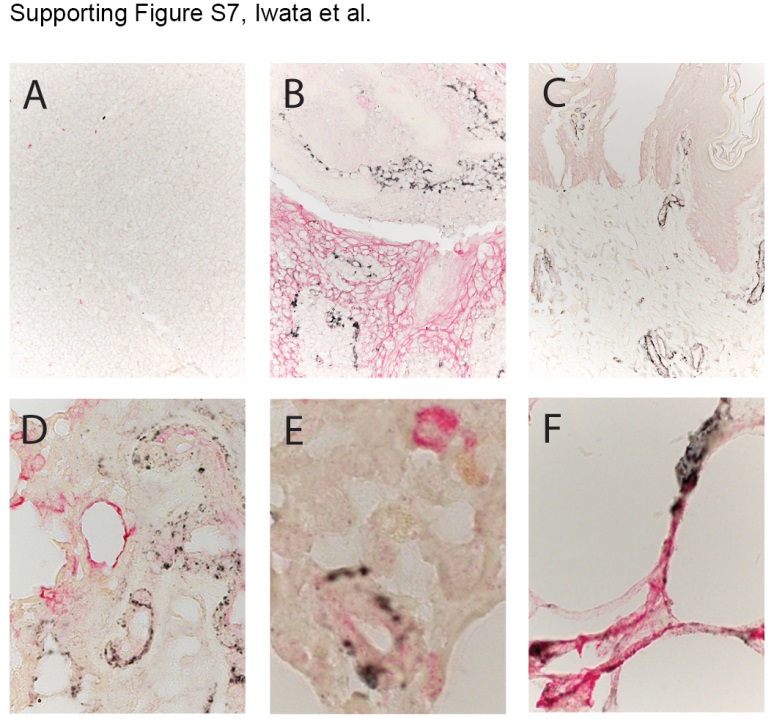


**Supporting Figure S7. Dual immunohistochemistry (IHC) of CDCP1 and CD146.** Tonsil (panels A and B), skin (panel C) and bone marrow biopsies (panels D-F) were stained with isotype-matched control antibodies (panel A) or antibodies against CDCP1 and CD146 (panels B-F). Positive cells were stained in red for CDCP1 and dark gray for CD146. Panels A-C: Positive and negative control staining show that epithelial cells in tonsil and epidermis are positive for CDCP1, whereas vascular endothelial cells are positive for CD146. Panel D: CDCP1+ cells (red) are clustered near fat cells and soma (left side of the panel) and CD146+ cells (dark gray) are localized in capillaries and sinusoids. Panels E: CD146+ cells (dark gray) surround a capillary (bottom) and a CDCP1+ cell (red) is present in soma (top). Panel F: Thin flattened CDCP1+ and CD146+ stromal cells surround fat cells. Original objective, X20 for panels A-D, X100 for panels E and F.
**Methods for Supporting Figure S7:** Dual IHC to detect CDCP1+ and CD146+ cells in marrow biopsies was performed at the Experimental Histopathology Laboratory at the FHCRC. Bone marrow biopsies were immediately fixed in 10% neutral buffered formalin, decalcified in Formical E (Fisher Scientific), then processed and embedded in paraffin. Sections (5 μm) were deparaffinized and rehydrated to distilled water. Heat-induced epitope retrieval was then performed with an EDTA based solution called Trilogy (Cell Marque, Rocklin CA) for 20 min in a steamer, followed by a 20-min cool down and incubated with 3% hydrogen peroxide for 8 min to block endogenous peroxidase activity. The sections were then incubated in Serum-free protein block (SFPB, Dako) solution containing 5% human serum for 10 minutes. They were incubated with a rabbit antibody against CD146 (Epitomics) for 1 hour. For a negative control, a concentration- and isotype-matched control was used. Slides were washed, incubated with secondary antibodies from an amplification kit (Dako) (CSA2RbtLink) for 15 min, washed again, and then incubated with a tertiary antibody (CSA2Amp, Dako) for 15 min. The slides were washed again and incubated with an additional reagent (CSA2FITC-HRP, Dako) for 15 minutes. The slides were then incubated in a chromogen (Deep Space Black) for 5 min. Another round of staining for CDCP1 was repeated. The slides were incubated with a rabbit antibody against CDCP1 (Cell Signaling) for 1 hour, washed, and then incubated with Leica’s PowerVIsion Rbt-AP for 30 min. The slides were incubated in a chromogen (Permanent Red, Dako) for 20 min, rinsed well with water, air dried and mounted with crystal mount. After the crystal mount cured at 37^o^C, the slides were rinsed briefly in xylene and coverslipped.


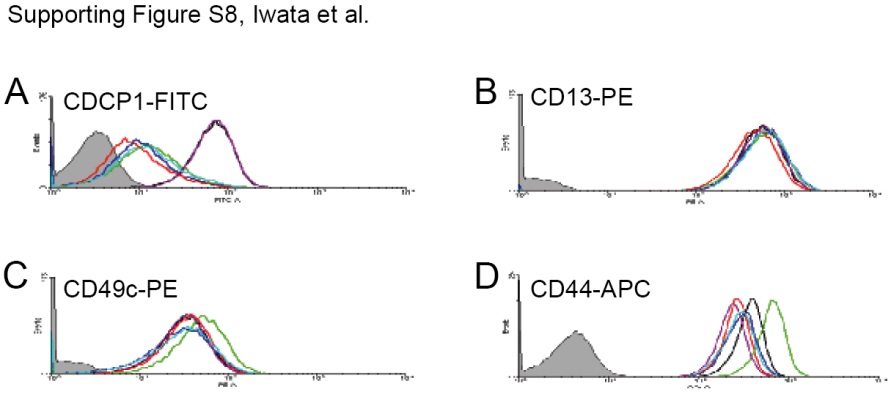


**Supporting Figure S8. Knock-down of CDCP1 by various siRNAs.** Surface protein expression of CDCP1 (Panel A), CD13 (Panel B), ITGA3/CD49c (Panel C) and CD44 (Panel D) was analyzed after transfecting various siRNAs for CDCP1 by flow cytometry. Four commercially available siRNAs for CDCP1 were used: Hs_CDCP1_2 (green line), HS_CDCP1-4 (dark blue line), Hs_CDCP1_5 (purple line), Hs_CDCP1_6 (red line), 4 siRNAs combined (light blue). Black line shows control cells transfected with siRNA for luciferase, and gray area indicates isotype-matched control for flow cytometry. One of the four siRNA, Hs_CDCP1_4 (dark blue line) showed significant inhibition of CDCP1 expression without changing surface expression of unrelated proteins, CD13, ITGA3/CD49c and CD44. Therefore, this siRNA was used for the subsequent experiments. The other siRNAs did not show the on target effect or showed obvious off target effects, and were not used in any further experiments, i.e. Hs_CDCP1_5 did not decrease CDCP1 expression, Hs_CDCP1_2 (green line) increased expression of ITGA3/CD49c and CD44, and Hs_CDCP1_6 (red line) decreased CD44 expression.
